# Design of a Bistable Artificial Venus Flytrap Actuated by Low Pressure with Larger Capture Range and Faster Responsiveness

**DOI:** 10.3390/biomimetics8020181

**Published:** 2023-04-26

**Authors:** Junchang Yang, Fenghui Wang, Yongjun Lu

**Affiliations:** Bio-Inspired and Advanced Energy Research Center, School of Mechanics, Civil Engineering and Architecture, Northwestern Polytechnical University, Xi’an 710129, China; jcyang@mail.nwpu.edu.cn (J.Y.); luyongjun@mail.nwpu.edu.cn (Y.L.)

**Keywords:** flytrap-inspired, composite structures, bistable, soft robots, dimensional optimization

## Abstract

The rapid closure of the Venus flytrap (*Dionaea muscipula*) can be completed within 0.1–0.5 s due to the bistability of hyperbolic leaves and the curvature change of midrib. Inspired by its bistable behavior, this paper presents a novel bioinspired pneumatic artificial Venus flytrap (AVFT), which can achieve a larger capture range and faster closure action at low working pressure and low energy consumption. Soft fiber-reinforced bending actuators are inflated to move artificial leaves and artificial midrib fabricated from bistable antisymmetric laminated carbon fiber-reinforced prepreg (CFRP) structures, and then the AVFT is rapidly closed. A two-parameter theoretical model is used to prove the bistability of the selected antisymmetric laminated CFRP structure, and analyze the factors affecting the curvature in the second stable state. Two physical quantities, critical trigger force and tip force, are introduced to associate the artificial leaf/midrib with the soft actuator. A dimension optimization framework for soft actuators is developed to reduce their working pressures. The results show that the closure range of the AVFT is extended to 180°, and the snap time is shortened to 52 ms by introducing the artificial midrib. The potential application of the AVFT for grasping objects is also shown. This research can provide a new paradigm for the study of biomimetic structures.

## 1. Introduction

Some organisms in nature can perform specific functions through particular movements to better survive in complex environments, and researchers can analyze and mimic the unique smart structures and efficient movement mechanisms of these organisms to achieve special functions and solve complex engineering problems. One of these organisms is the Venus flytrap (*Dionaea muscipula*), a carnivorous plant known as “one of the most wonderful plants in the world” [1] that captures prey with a trap formed by a pair of symmetrical leaves. When prey touches the trigger hairs located on the inner surface of the leaves twice within a certain period, the action stimulates rapid closure of the leaves within 0.1–0.5 s [2], finally forming an inescapable cage together with the spiky hairs on the outer edge of the leaves. Forterre [3] was the first to explain the closure mechanism of the Venus flytrap on the macroscopic scale, i.e., the flytrap mainly utilizes the snap-buckling instability of its hyperbolic leaves to perform rapid closure, and divided the closure process into three phases: a slow initial phase, a rapid intermediate phase, and finally, a second slow phase. The slow initial phase is an active biochemical process in which the flytrap will generate action potentials after being stimulated to “actively” change the curvature of leaves and store elastic potential energy in the leaves. The rapid intermediate phase and the second slow phase are passive elastic processes, with most of the displacement of the leaves occurring in the intermediate phase, when the flytrap releases its previously stored elastic potential energy to change the shape of the leaves from convex to concave. However, there are few reports about the second slow phase of the closure process. Considering the composition of the midrib, it is reasonable to speculate in this paper that the midrib undergoes deformation during the second slow phase. Specifically, the midrib is responsible for connecting two leaves and consists of a large number of cells with non-negligible stiffness and thickness. Therefore, one of the necessary conditions for the perfect closure of the Venus flytrap is that the midrib gradually decreases in thickness or increases in curvature at the second slow phase, which inspired us to introduce an artificial midrib into the artificial Venus flytrap (AVFT) to simulate the closure of flytrap more realistically.

With the development of materials science, researchers have used the dynamic properties of some special materials to develop a number of systems to imitate the closure action of a Venus flytrap [4]. Esser, et al. [5] summarized recent developments in flytrap-inspired soft machine systems based on movement principles while using harvesting and storing energy from the environment, and sensing information as criteria for distinguishing the AVFTs from the real Venus flytrap. Various types of smart materials as main part or driving part are key for AVFTs to acquire the ability to respond to environmental stimuli or make fast closure movements, and these smart materials include carbon fiber-reinforced prepreg (CFRP) [6,7,8,9,10], hydrogels [11,12], silicone rubber [13], liquid crystal elastomers (LCEs) [14], shape memory alloys (SMAs) [6,7,8], electroactive polymers [15,16,17,18], polydimethylsiloxane (PDMS) [19], metamaterials [20] and so on. It is worth mentioning that, on the basis of a compliant foil with a rigid backbone [21], Tauber, et al. [22] can use the various above-mentioned materials to realize the snap buckling and environmental response of the AVFT, such as rubber, SMAs, magnets and hydrogels. Composite laminates with two different stable states are called bistable composite structures, and their snap process has remarkable similarity with the capture process of the Venus flytrap. Compared with other smart materials, the bistable composite structures have higher critical strain energy. In addition, the bistable CFRP structure has a more mature manufacturing process compared with other bistable composite structures. Therefore, the AVFT tends to obtain faster closure speed if the bistable CFRP structures are used as its main part. The particular bistable mechanism of CFRP structures has been extensively studied [23,24,25,26,27,28,29,30,31,32] and applied in the AVFT. Kim [6,7,8] integrated a shape memory alloy into a general asymmetric orthogonal laminated CFRP structure to create a AVFT where the artificial leaves can be automatically closed and opened by controlling the temperature, and the closure time was controlled to within 100 ms. Like the general asymmetric orthogonal laminated CFRP bistable structures, the antisymmetric laminated CFRP structures also have excellent bistable behavior, but require a larger driving force to trigger the snap process. Zhang utilized the attractive force [9] and repulsive force [10] of the magnets to drive the artificial leaves made of antisymmetric laminated CFRP structure, which was mechanically designed to achieve non-contact control with excellent responsiveness. However, there is a problem with the above AVFTs. Both of their main parts consist of only two artificial leaves, which results in small closure range and slow closure speed. Inspired by the midrib of Venus flytrap, we introduced an artificial midrib into the AVFT, which enabled the AVFT to obtain larger closure range and faster closure speed.

As a soft robotic gripper, the AVFT based on the bistable CFRP structure exhibits sufficient flexibility to adapt to the deformation of objects [10,33]. Fluidic elastomer actuators are one of the oldest but still widely used soft robot actuation technologies [34], with advantages such as easy fabrication, low cost, robustness, fast response time and easy implementation [35,36] compared to shape memory material actuation [37,38,39,40,41], magnetic actuation [42,43] and other actuation methods [44,45,46]. Soft pneumatic bending actuators are typically representative of pneumatic actuations, which achieve bending deformation via a flexible asymmetrical axial cross-section surrounding an inflatable void. Three types of axial asymmetry have been developed for soft pneumatic bending actuators [47]: multi-material [48,49,50,51,52,53], corrugated membranes [54,55,56,57,58,59,60] and eccentric void asymmetries [61,62]. It is worth mentioning that the soft pneumatic bending actuator can not only be fabricated by traditional cast molding methods, but can also be fabricated by 3D/4D printing technologies benefiting from the recent advances in additive manufacturing technology [57,60,63]. In general, most soft pneumatic bending actuators can only provide limited tip force. The typical representative of multi-material soft pneumatic bending actuators, the soft fiber-reinforced bending actuator, can withstand higher working pressure to generate higher tip force [64], which can generate sufficient driving force to trigger the snap-through behavior of the bistable CFRP structure by adjusting the geometrical parameters. However, high working pressure means that more energy input is required, which can lead to the failure of the soft fiber-reinforced actuator and bring safety risks. Therefore, the focus of this paper is to optimize the dimensions of the soft fiber-reinforced bending actuator, so that it can accomplish the actuation of the bistable CFRP structure at low working pressure.

In this paper, we demonstrate a novel pneumatic bioinspired AVFT based on bistable deformable structures. The main part of AVFT consists of one artificial midrib and two artificial leaves, both of which are made of bistable antisymmetric laminated CFRP structures. The introduction of the artificial midrib enables AVFT to achieve up to 180° capture range and snap time as low as 52 ms compared to previous AVFTs consisting of only two artificial leaves. In addition, we design a novel low-cost experiment to measure the critical triggering force of the antisymmetric laminated CFRP structures, which does not require expensive experimental equipment or complex experimental steps. A dimensional optimization framework for soft fiber reinforced actuators is developed using the Pointer algorithm to reduce their working pressures.

The rest of the paper is organized as follows. In Section 2, we use a two-parameter model to explore whether the selected antisymmetric laminated CFRP structure has bistable behavior, analyze the factors affecting the longitudinal curvature of structure in the second stable state and predict the curvature while verifying it using a finite element model. In Section 3, combining the above theoretical conclusions, we describe the design concept of AVFT in detail. In Section 4, the principle of the new experiment is explained, and the critical trigger forces for the snap process of bistable CFRP structures are measured and compared with numerical results. In Section 5, the minimum working pressure required when the tip force of the soft actuator reaches the critical trigger force of the artificial leaf or artificial midrib is taken as the optimization function, and the geometry parameters of soft actuators are optimized to reduce the working pressure based on the finite element model of the tip force, and the experimental validation is carried out. In Section 6, we obtain the driving time and snap time of AVFT with a high-speed camera. The potential application of the AVFT as a soft gripper for grasping objects is also shown.

## 2. Curvature Analysis for the Bistable Antisymmetric Laminated CFRP Structure

**Figure 1:**
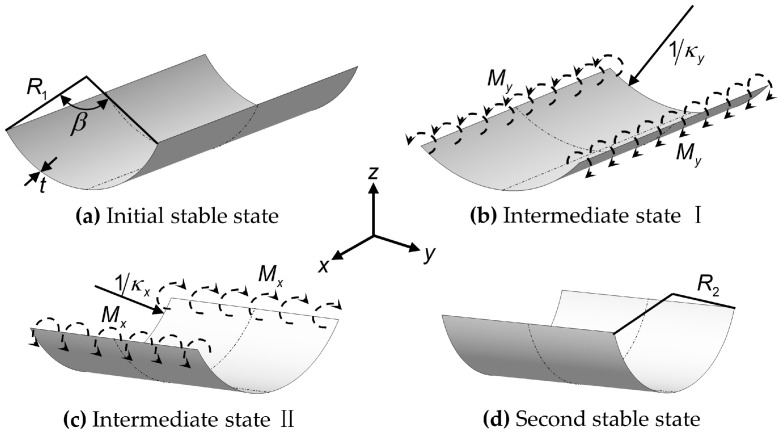
Snap process of the bistable antisymmetric laminated carbon fiber-reinforced prepreg (CFRP) structure. (**a**,**d**) show the structure in the initial and second stable state, respectively, which can be switched by applying bending moments to the two straight edges of the structure. *R*_1_, *β*, *R*_2_ and *t* indicate the radius and central angle of the cross-section arc of the structure in the initial stable state, the radius of the cross-section arc of the structure in the second stable state, and the thickness of the structure, respectively. (**b**,**c**) show the two intermediate states of the snap process of structure, which can be described by transverse curvature $\kappa_{y}$ and longitudinal curvature $\kappa_{x}$.

The antisymmetric laminated structure is composed of some single-layer plates of the same size, and the fiber layout of these single-layer plates is antisymmetric with respect to the mid-surface, which can exhibit interesting bistable behavior similar to that of the Venus flytrap under certain conditions. Figure 1 shows a schematic diagram of the snap process of the bistable antisymmetric laminated CFRP structure, and the *x* and *y*-axes correspond to the longitudinal and transverse directions of the structure. The two stable states of the structure can be regarded as a cylindrical shell with thickness *t* (Figure 1a,d). In the initial stable state, the radius of the cross-section arc is *R*_1_, and the subtended angle is *β*. In the second stable state, the radius of the cross-section arc is *R*_2_. Note that the centers of curvature of the cross-sections in the two stable states are on the same side of the structure. The two stable states of the structure correspond to two local minima of the strain energy, respectively. The strain energy can be stored by applying bending moments to the two straight edges of the structure. Once the strain energy exceeds the potential energy barrier, the structure will enter the snap process and finally complete the stable state transition. Figure 1b,c show the two intermediate states of the snap process of structure, which can be described by transverse curvature $\kappa_{y}$ and longitudinal curvature $\kappa_{x}$. The bistable behavior of antisymmetric laminated CFRP structure is generally related to material properties, geometric configurations, and lay-up methods. Therefore, using the theoretical model to determine whether the structure has bistability, to explore the factors affecting the curvature in the second stable state, and predict the curvature in the second stable state, is of guiding significance for the design of the AVFT.

The single-layer plate is orthotropic material because it has two orthogonal planes of elastic symmetry. According to the Classical Lamination Theory, the ***ABD*** matrix for the antisymmetric CFRP structure laminated by the CFRPs under plane stress is

(1) $$\left[\begin{array}{c} N\\ M \end{array}\right]=\left[\begin{array}{c} N_{x}\\ N_{y}\\ N_{xy}\\ M_{x}\\ M_{y}\\ M_{xy} \end{array}\right]=\left[\begin{array}{cccccc} A_{11} & A_{12} & 0 & 0 & 0 & B_{16}\\ A_{12} & A_{22} & 0 & 0 & 0 & B_{26}\\ 0 & 0 & A_{66} & B_{16} & B_{26} & 0\\ 0 & 0 & B_{16} & D_{11} & D_{12} & 0\\ 0 & 0 & B_{26} & D_{12} & D_{22} & 0\\ B_{16} & B_{26} & 0 & 0 & 0 & D_{66} \end{array}\right]\left[\begin{array}{c} \varepsilon_{x}^{0}\\ \varepsilon_{y}^{0}\\ \gamma_{xy}^{0}\\ \kappa_{x}\\ \kappa_{y}\\ \kappa_{xy} \end{array}\right]=\left[\begin{array}{cc} A & B\\ B & D \end{array}\right]\left[\begin{array}{c} \varepsilon^{0}\\ \kappa \end{array}\right],$$

where ***N***, ***M*** are the internal load and moment, respectively, $\varepsilon^{0}$ is the mid-plane strain associated with moving from the initial stable state to any intermediate state, κ is the corresponding mid-surface curvature, *A*_ij_, *B*_ij_ and *D*_ij_ represent the stretching stiffness, coupling stiffness and bending stiffness of the structure, respectively. The bending strain energy *U_b_* and the stretching strain energy *U_s_* for a unit length of structure can be obtained from the following two equations respectively [28] (see Note 1 in Supplementary Information S1 for more theoretical derivation details, including Figure S1):

(2) $$U_{b}=\frac{\beta R_{1}}{2}\left[D_{11}\kappa_{x}^{2}+2D_{12}\kappa_{x}\left(\kappa_{y}-\frac{1}{R_{1}}\right)+D_{22}{\left(\kappa_{y}-\frac{1}{R_{1}}\right)}^{2}\right],$$

(3) $$U_{s}=\left\{\begin{array}{ll} \frac{A_{11}}{2}\left[\frac{\beta R_{1}}{2}\left(\frac{\kappa_{x}^{2}}{\kappa_{y}^{2}}\right)+\frac{\sin\left(\beta R_{1}\kappa_{y}\right)}{2}\left(\frac{\kappa_{x}^{2}}{\kappa_{y}^{3}}\right)-\frac{4\sin^{2}\left(\beta R_{1}\kappa_{y}/2\right)}{\beta R_{1}}\left(\frac{\kappa_{x}^{2}}{\kappa_{y}^{4}}\right)\right] & \left(\kappa_{y}\neq 0\right)\\ 0 & \left(\kappa_{y}=0\right) \end{array}\right..$$

Neglecting the stretching-bending coupling effect, the total strain energy in a unit length of the antisymmetric laminated CFRP structure is the sum of the bending strain energy and the stretching strain energy, that is,

(4) $$U=U_{b}+U_{s}.$$

If the lay-up method and material properties are known, the total strain energy of the structure is determined by only two parameters, $\kappa_{x}$ and $\kappa_{y}$. Equation (4) is the two-parameter model for the antisymmetric laminated CFRP structure proposed by Iqbal and Pellegrino [28], which can also be called the extensional bending model [32]. It is known that the transverse curvature $\kappa_{y}$ of the structure in the second stable state is equal to 0. According to the principle of minimum potential energy, by making the first derivative of the total strain energy *U* with respect to the longitudinal curvature $\kappa_{x}$ equal to 0, the expression for the longitudinal curvature $\kappa_{x}$ of the antisymmetric laminated CFRP structure in the second stable state can be obtained:

(5) $$\kappa_{x}=\frac{D_{12}}{D_{11}}\cdot \frac{1}{R_{1}}.$$

The above equation shows that the longitudinal curvature $\kappa_{x}$ of the structure in the second stable state is related to the radius of transverse curvature *R*_1_ in the initial stable state, the material properties of the CFRPs and the lay-up methods, but has no correlation with the length of the edges of the structure and the central angle subtended by the cross-section arc in the initial stable state.

The laminate made of T800/924C epoxy resin-based CFRPs with a ply thickness of 0.125 mm and the lay-up [45°/−45°/45°/−45°] are selected as the research object of this paper. The material properties of the CFRP are shown in Table 1. The transverse radius of curvature *R*_1_ of the structure in the initial stable state is 47.75 mm, and the central angle *β* is π.

The ***ABD*** matrix of the selected antisymmetric laminated CFRP structure is

(6) $$\left[\begin{array}{cc} A & B\\ B & D \end{array}\right]=\left[\begin{array}{cccccc} 22.65 & 17.65 & 0 & 0 & 0 & 0\\ 17.65 & 22.65 & 0 & 0 & 0 & 0\\ 0 & 0 & 18.70 & 0 & 0 & 0\\ 0 & 0 & 0 & 0.47 & 0.37 & 0\\ 0 & 0 & 0 & 0.37 & 0.47 & 0\\ 0 & 0 & 0 & 0 & 0 & 0.39 \end{array}\right],$$

where the units are GPa·mm for stretching stiffness ***A***, GPa·mm^2^ for coupling stiffness ***B***, and GPa·mm^3^ for bending stiffness ***D***. The coupling stiffness ***B*** = 0 in the above equation indicates that there is no coupling effect of stretching and bending for the structure with the lay-up [45°/−45°/45°/−45°]. Also note that *D*_16_ = *D*_26_ = 0, i.e., bending and twisting are decoupled, which indicates that the longitudinal and transverse directions of curvature of the structure in the initial stable state will be principal directions of curvature also in the second stable state.

Pellegrino presented a stability criterion in order to determine the existence of bistability for structures with no coupling between bending and twisting:

(7) $$S=4\frac{D_{66}}{D_{11}}+2\frac{D_{12}}{D_{11}}-2\frac{D_{22}}{D_{12}},$$

if *S* > 0, the structure has bistable behavior [32]. It is easy to obtain the *S* = 2.353 > 0 for the selected antisymmetric laminated CFRP structure, which has bistable behavior.

**Figure 2:**
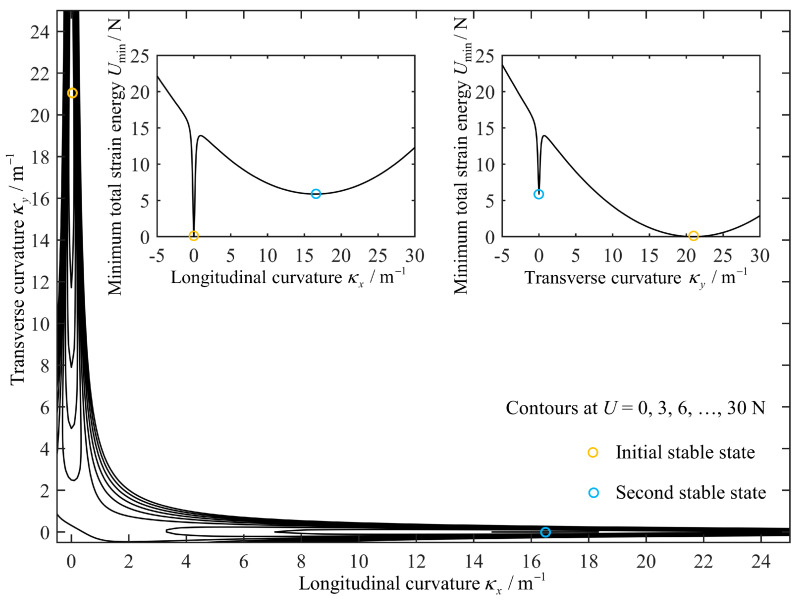
The total strain energy *U* per unit length of the selected antisymmetric laminated CFRP structure. The inset graphs are the relationship between longitudinal/transverse curvature and minimum total strain energy.

According to Equation (4), a contour plot of the total strain energy *U* per unit length of the selected antisymmetric laminated CFRP structure as a function of transverse curvature $\kappa_{y}$ and longitudinal curvature $\kappa_{x}$ can be obtained, as shown in Figure 2. Then, taking the longitudinal curvature $\kappa_{x}$ as any constant value in the interval [−5, 30], the minimum value of the total strain energy *U*_min_ for the transverse curvature $\kappa_{y}$ in the interval [−5, 30] can be determined, and the variation of *U*_min_ with $\kappa_{x}$ is shown in the left graph inside Figure 2. In the same way, the change of *U*_min_ with $\kappa_{y}$ can also be obtained, as shown in the right graph inside Figure 2.

Figure 2 clearly shows the existence of two local minima in the total strain energy per unit length of antisymmetric laminated CFRP structure, corresponding to two stable states, where the position of the initial stable state is marked by the yellow circle and the second stable state is marked by the blue circle. This can be clearly verified in the inset graphs: *U*_min_ corresponding to $\kappa_{x}=0$ in the left graph is the first local minimum of total strain energy, and the corresponding transverse curvature $\kappa_{y}$ is equal to 20.94 m^−1^ through calculation; Similarly, *U*_min_ corresponding to $\kappa_{y}=20.94{\;m}^{-1}$ in the right graph is also a local minimum of total strain energy, which is equal to the first local minimum of total strain energy, and the corresponding longitudinal curvature $\kappa_{x}$ is equal to 0 through calculation. In fact, the two local minima correspond to the same stable state of the structure—the initial stable state (marked by the yellow circle), where $\kappa_{x}=0$, $\kappa_{y}=20.94{\;m}^{-1}$ and *U*_min_ = 0. In the same way, the theoretical predictions for the second stable state of selected structure can be obtained (marked by the blue circle), namely $\kappa_{x}=16.49{\;m}^{-1}$, $\kappa_{y}=0$ and *U*_min_ = 5.9 N. In addition, the curvature change follows the minimum energy path, i.e., it only changes within a narrow “L-shaped strip” where the Gaussian curvature is approximately equal to 0.

**Figure 3:**
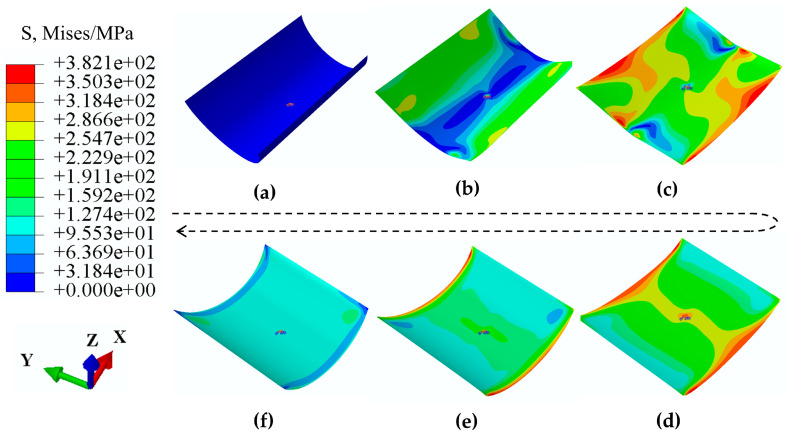
Finite element analysis for snap process of the selected bistable antisymmetric laminated CFRP structure. (**a**,**f**) show the structure in the initial and second stable state, respectively, and (**b**–**e**) show the intermediate states in the snap process of structure.

In order to verify the reliability of the curvature predicted by the theoretical model, a finite element analysis was performed using Abaqus (Dassault Systèmes, Aachen, Germany) for the selected antisymmetric laminated CFRP structure (see Note 2 in Supplementary Information S1 for more simulation details). Figure 3 shows the numerical result of the snap process of the selected bistable antisymmetric structure, where Figure 3a,f correspond to the initial stable state and second stable state of the structure, respectively. Note that the stress field still exists when the structure is in the second stable state, although the load has been unloaded, which is consistent with the theoretical prediction. Converting the .inp file containing the coordinates of each node of the deformed structure into a .stp file and importing it into Catia, then using Porcupine Curvature Analysis to obtain the longitudinal curvature $\kappa_{x}$ of the selected antisymmetric laminated CFRP structure as 15.22 m^−1^, which is very close to the theoretical prediction value.

## 3. Design Concept of the AVFT

The bistability of the CFRP structure makes it one of the most suitable materials for fabricating the AVFT. Previous researchers have only focused on imitating the closure action of the leaves, paying no attention to the effect of the midrib in the closure process of Venus flytrap [6,7,8,9,10]. On the one hand, adding an artificial midrib can more accurately simulate the real Venus flytrap from a bio-inspired point of view. On the other hand, the artificial midrib acts as a transition section connecting the two artificial leaves, increasing the capture range of the AVFT and reducing its closure time., which will be demonstrated in Section 3 and Section 6.

**Figure 4:**
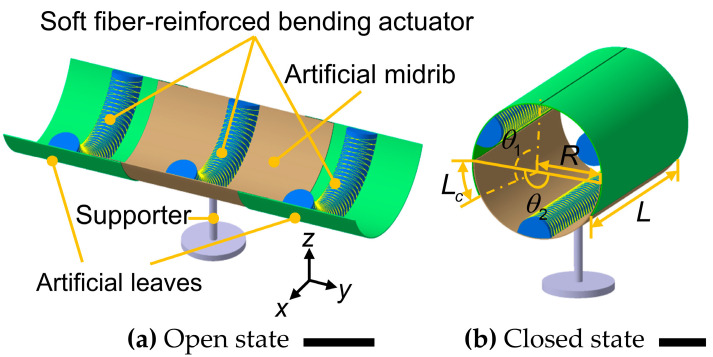
Prototype of the Artificial Venus flytrap (AVFT) in the (**a**) open and (**b**) closed states. Scale bars, 50 mm.

The main part of the AVFT is designed through a reverse-design strategy. That is, the dimensions of the artificial midrib and artificial leaves are reasonably designed firstly to make the AVFT close seamlessly. Both the artificial leaves and the artificial midrib are made of bistable antisymmetric laminated CFRP structures (see Note 3 in Supplementary Information S1 for more fabrication details, including Figure S2), where the artificial midrib is the same as the structure selected in Section 2. Figure 4b shows the AVFT in the closed state, which is presented as a thin cylindrical shell with an axis length *L* = 150 mm and a radial cross-sectional radius *R* = 150/π mm, at this time the artificial leaves and the artificial midrib are in the initial stable state. The central angles of the artificial leaves and the artificial midrib are $\theta_{1}$ = 120° and $\theta_{2}$ = 180°, respectively. VHB^TM^ 5952 double-sided tape (3M, St. Paul, MN, USA) is chosen to fix two artificial leaves on the outside of the artificial midrib with the arc length of the overlap area *L_c_* = 25 mm. Only one screw exists throughout the AVFT, located in the center of the artificial midrib, which is used to fasten the AVFT to a supporter. Due to the holes are drilled into the composite structure before placing the screws, cracks can easily form around the holes, which can lead to crack extension over time. Therefore, the fixation method used in this work can improve the service life to some extent compared to past CFRP-based AVFTs that used multiple screws. Next, we manually switched the AVFT from the closed state to the open state; at this time, the artificial leaves and the artificial midrib are in the second stable state, as shown in Figure 4a. Since the artificial leaves and the artificial midrib are made of the same material and the same lay-up method, and have the same transverse curvature in the initial stable state, according to the conclusion in Section 2, they have the same longitudinal curvature in the second stable state, which will make the artificial leaves and the artificial midrib closely fit in the overlapping area, and ensure the continuous deformation of the overlapping area during the snap process. It is worth noting that the artificial midrib, as the connecting part of the two artificial leaves, has a curvature of 0 on the *y*-axis, which enables the AVFT to obtain larger capture range—up to 180°.

**Figure 5:**
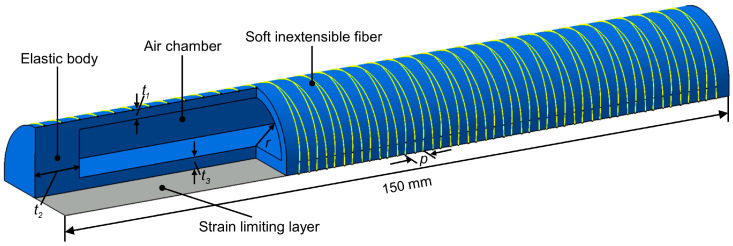
The structure of soft fiber-reinforced bending actuator consists of an elastic body, flexible inextensible fibers, a strain-limiting layer and an air chamber, which can be modelled by the four geometric parameters *r*, *t*_1_, *t*_2_, *t*_3_, and *p*.

Three soft fiber-reinforced actuators as driving parts are attached to the middle of the artificial leaves and artificial midrib by VHB^TM^ 5952 double-sided tape. The soft fiber-reinforced actuator consists of an elastic body, flexible inextensible fibers, a strain-limiting layer and an air chamber. Its mode of motion, including bending, twisting and extending, can be controlled by changing the winding of the flexible inextensible fibers and the length of the strain-limiting layer [49]. Obviously, using a configuration that allows the fiber-reinforced actuator to bend is most appropriate for triggering the deformation of the artificial leaves and the artificial midrib. As shown in Figure 5, a strain-limiting layer is affixed to the plane of the elastic body to limit the extension of the plane during inflation, and flexible inextensible fibers are wrapped around the surface of the elastic body in a symmetrical double helix structure to limit its radial expansion. The soft actuator fabricated from a high-hardness silicone rubber material can apply higher force to objects [55]. To obtain sufficient driving force to actuate the bistable antisymmetric laminated CFRP structure with a layer thickness of 0.5 mm, we chose Smooth-Sil 950 (Smooth-on, Inc., Macungie, PA, USA) with a hardness of 50 A for the elastic body. The cross-section of the elastic body with the internal cavity is designed as a semicircle, because it can produce the same bending moment as a rectangular cross-section and is easier to bend when the same working pressure is provided [51] (see Note 4 in Supplementary Information S1 for more fabrication details). In addition, the qualitative analysis by equating the soft actuator to an orthotropic single-layer plate shows that the addition of the soft actuator slightly increases the stretching and bending stiffness of the system, and causes the coupling effect between stretching and bending in the system.

In summary, the AVFT consists of two artificial leaves, one artificial midrib, one supporter and three soft fiber-reinforced bending actuators. When the AVFT is open with a capture range of 180° (Figure 4a), increasing the working pressure of three soft actuators generates the force perpendicular to the surface of the artificial leaves and artificial midrib. As the working pressure increases, the increasing force gradually flattens the surface. Eventually, the artificial leaves and artificial midrib snap rapidly and automatically to the initial stable state, at which point the AVFT is completely closed (Figure 4b). It is worth noting that after releasing the inner air of the soft actuators, the AVFT remains closed without additional energy input due to the bistability of the main part.

## 4. Experiment of the Critical Trigger Forces for Artificial Leaves and Artificial Midrib

**Figure 6:**
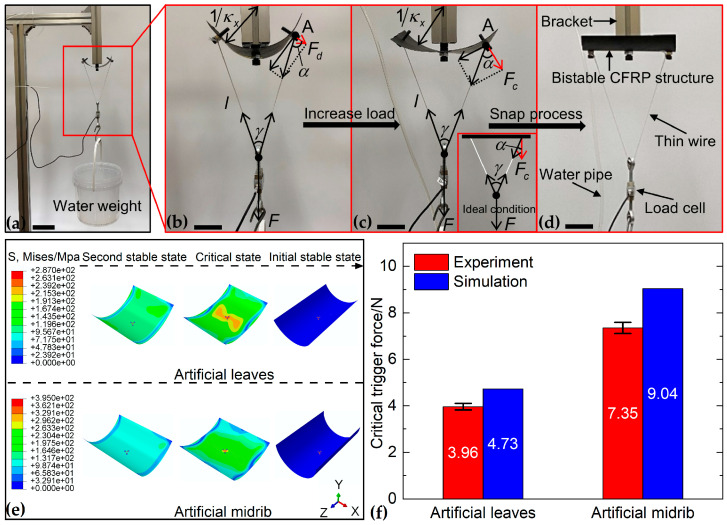
(**a**) The experiment of critical trigger force of bistable antisymmetric laminated CFRP structure. (**b**) As the load increases, the tension of the thin wire increases and the driving force at the bolt also increases so that the surface of the structure is gradually unfolded. (**c**) When the driving force reaches a critical point, the bistable CFRP structure changes from the second stable state to (**d**) the initial stable state through snap process. The lower right-hand corner of (**c**) shows the hypothetical critical state. $F_{d}$, $F_{c}$, F, α, κ, γ and l indicate the driving force, the critical trigger force, the load, the angle formed by the normal force and tangential force, the curvature of structure, the angle formed by the thin wire and half the length of the thin wire, respectively. (**e**) Snap process of artificial leaves and artificial midrib obtained by finite element analysis. (**f**) Comparison of the critical trigger forces between experimental and numerical results. Error bars represent the standard deviation (n = 3). Scale bars, 180 mm in (**a**) and 40 mm in (**b**–**d**).

In previous studies, the critical trigger force can be measured by using a constant pulley mechanism, a linear motor or a universal tensile testing machine [7,8,66,67]. However, these experimental methods require expensive experimental equipment or complex experimental steps. We designed a simple experiment, as shown in Figure 6a. A T-shaped aluminum bracket was fixed to the floor. When the structure was in the second stable state, two holes were punched, and bolts were attached near the middle of the straight edge of the structure. The center of the structure was also drilled and bolted to the T-shaped aluminum bracket. A thin wire was then passed through the left bolt, the load cell (CHINO SENSOR Co., Ltd., Bengbu, China) and the right bolt in turn. A container was hung under the load cell, and water was fed into the container as a load through a rigid water pipe with an internal diameter of 4 mm to ensure sufficient water pressure for uniform loading.

The small-diameter water pipe limits the speed of load increase, so the whole loading process of this experiment can be considered a quasi-static process. As shown in Figure 6b, taking point A as an example, the pulling force of the thin wire on point A can be broken down into a normal force and a tangential force on the surface of the bistable CFRP structure. The tangential force can form a bending moment, which is ignored because of the small distance between the wire and the surface. The normal force is therefore called the driving force, and it is equivalent to the tip force exerted by the soft actuator. The critical trigger force is the minimum driving force that causes the bistable CFRP structure to snap. As the load increases, it can be seen that the curvature of the bistable CFRP structure gradually decreases. We assume that the bistable CFRP structure has the same curvature at all points, and the curvature approaches 0 in the critical state. Thus, we can obtain the relationship between the driving force and the load:

(8) $$\begin{array}{ll} F_{d} & =\frac{F}{2\cos\frac{\gamma }{2}}\cdot \cos\alpha \\ & =\frac{F}{2\cos\frac{\gamma }{2}}\cdot \cos\left[\arcsin\left(l\kappa_{x}\sin\frac{\gamma }{2}\right)+\frac{\gamma }{2}\right] \end{array},$$

where $F_{d}$, F, α, κ, γ and l is the driving force, the load, the angle formed by the normal force and tangential force, the curvature of structure, the angle formed by the thin wire and half the length of thin wire, respectively. Figure 6c is the critical state of the bistable CFRP structure before the snap process. According to the assumption, at this time κ→0, we can conclude:

(9) $$F_{c}=F_{d}=\frac{F}{2},$$

where $F_{c}$ is the critical trigger force. Thus, the tip force when the bending angle of the soft actuator is 0° larger than half of the load can drive the deformation of the bistable antisymmetric laminated CFRP structure.

We prepared three samples for both artificial leaf and artificial midrib. The critical trigger force was measured three times for each sample, the testing data were averaged, and the standard deviation calculated. The results are shown in Figure 6f; the soft actuators need to generate the tip force of 3.96 N and 7.35 N, respectively, at a bending angle of 0° to drive the artificial leaves and artificial midrib from the second stable state to the initial stable state. We used Abaqus to simulate the snap process of the artificial leaves and the artificial midrib with the same boundary conditions as the experiment to obtain the numerical results of the critical trigger force, as shown in Figure 6e (see Note 5 in Supplementary Information S1 for more simulation details). The numerical results for the critical trigger force, shown in Figure 6f, are 4.73 N and 9.04 N, respectively. The difference between the viscoelastic behavior of epoxy resin and the finite element model based on the assumption of linear elasticity leads to some errors.

## 5. Optimization of the Working Pressures for Soft Fiber-Reinforced Bending Actuators

### 5.1. Optimization Framework and Results

**Figure 7:**
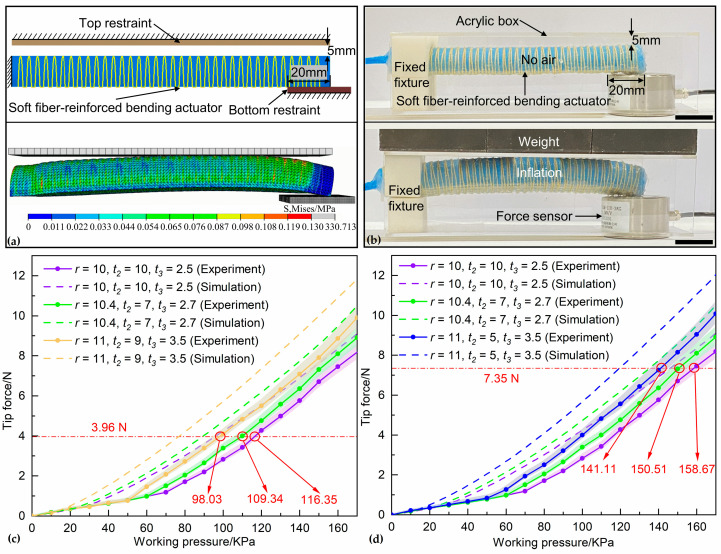
(**a**) Simulation and (**b**) experiment of the tip force of the soft actuator, the upper picture shows the positional relationship of each component, and the lower picture shows the deformation state after inflation. (**c**,**d**) show the relationship between the tip force and working pressure of selected soft actuators, including experimental and numerical results, which demonstrate the reliability of the optimized results. Shaded areas represent the standard deviation (n = 3). Scale bars, 20 mm.

A finite element model was built to prepare for dimension optimization of the soft fiber-reinforced bending actuators. We use Abaqus to obtain a numerical relationship between the tip force generated by the soft fiber-reinforced bending actuator at a bending angle of 0° and the working pressure. As shown in the upper part of Figure 7a, the model consists of a soft actuator, a top restraint and a bottom restraint. The distance between the top restraint and the top of the soft actuator is 5 mm, and the bottom restraint is in contact with the distal end of the soft actuator. Uniaxial tensile tests were carried out to determine the parameters of the hyperelastic constitutive model of the silicone rubber (Smooth-Sil 950) used to make the elastic body of the soft actuator according to ASTM D638 [54,68]. After using least squares curve fitting to determine the coefficients, we found that the second-order polynomial model fits the experimental data best with the coefficients determined as *C10* = −0.01198, *C20* = −0.004238, *C01* = 0.1305, *C02* = 0.2294 and *C11* = −0.004517 (see Note 6 in Supplementary Information S1 for more simulation details, including Figure S3). The bottom part of Figure 7a shows the deformation state of the soft actuator after inflation obtained by simulation.

The tip force of the soft fiber-reinforced bending actuator varies with the geometrical parameters. To fully optimize the dimensions of the soft actuator, five geometrical parameters are imported to parameterize the design of the soft actuator (Figure 5), including the chamber radius *r*, the wall thickness *t*_1_, the cap thickness *t*_2_, the base layer thickness *t*_3_ and the fiber pitch *p*. The length of the actuator was set to 150 mm to maximize the driving force and match the axis length of the bistable CFRP structure in the initial stable state. We used Isight (Dassault Systèmes, Aachen, Germany) integrated with Catia (Dassault Systèmes, Aachen, Germany), Abaqus and MATLAB (MathWorks, Inc., Natick, MA, USA) for optimization. The geometrical parameters *r*, *t*_1_, *t*_2_, *t*_3_ and *p* are imported into Isight as input parameters. Isight automatically changes the values of the input parameters and then causes Catia to update the geometrical model through a batch program. The updated geometrical model is automatically imported into Abaqus for solving and outputting the numerical relationship between the tip force and the working pressure, which is then imported into MATLAB to obtain the working pressure required when the tip force equals the critical trigger force. Finally, the minimum working pressure is found by iterating the values of the geometric parameters, and the optimum geometric parameters are returned.

To improve the optimization efficiency, it is necessary to find the geometrical parameter that has a minor effect on the optimization objective among the five geometrical parameters. A parameter study is a method within the Design of Experiments (DOE) component that investigates the sensitivity of each parameter to the response independently of all other parameters by means of a controlled variables approach. Therefore, we can obtain the contribution of each of the geometrical parameters to the optimization objective. The range of the input parameters *r*, *t*_1_, *t*_2_, *t*_3_ and *p* were set as [8, 11], [1, 3], [5, 15], [1.5, 3.5] and [3, 5], respectively, with a level of 5. The initial values were set as 9.5 mm, 2.0 mm, 10 mm, 2.5 mm and 4 mm. Isight built a multiple linear regression model based on the above 25 sets of sample points and the corresponding responses, then transformed the normalized geometric parameter coefficients into percentage contribution rates. Finally, a pareto graph was obtained considering only the linear main effects of the input parameters (Figure S4). The pareto graph reflects the percentage contribution of the five geometric parameters to the working pressure, with blue indicating positive effects and red negative effects. It can be seen that the working pressure decreases as the parameters *r*, *t*_1_ and *t*_3_ increase, with *r* making the largest contribution of approximately 66.45% and *t*_1_ approximately 16.77%. Increasing *t*_2_ and *p* will increase the working pressure, with the contribution of *p* is minimal at only 1.01%. This indicates that the linear effect of the fiber pitch *p* to the working pressure is minimal. By weighing the difficulty of making the real soft actuator and its positive correlation with the working pressure, we set the fiber pitch *p* to 4 ∈ [3, 5] to simplify the optimized model. 

Pointer Automatic Optimizer algorithm [69] combines four optimization algorithms, including the linear simplex method, sequential quadratic programming, downhill simple method and genetic algorithms, which can be automatically grouped to form an optimal optimization strategy based on the results of each iteration, and has been found effective for solving design optimization of soft corrugated membranes bending actuator [70,71]. The maximum width of the soft actuator was set to 24 mm to reduce the effect on the initial curvature of the bistable CFRP structure. Therefore, the number of input parameters was reduced to three by adding a constraint *r* + *t*_1_ = 12 mm. We set the ranges of input parameters *r*, *t*_2_ and *t*_3_ as [9, 11], [5, 15] and [1.5, 3.5], respectively, with an increment of 0.1. The initial values were set as 10 mm, 10 mm and 2.5 mm. Both the dimensions of soft actuator required for the artificial leaves and the artificial midrib were optimized for 38 iterations. Figure S5a,b show the optimization of the dimensions of the soft actuator for the artificial leaves to minimize the required working pressure for a tip force equal to 3.96 N, starting at 95.49 KPa and ending at 78.11 KPa. The optimum dimensions were found for *r* = 11 mm, *t*_2_ = 9 mm and *t*_3_ = 3.5 mm, corresponding to a minimum working pressure of 76.56 KPa. Figure S5c,d show the optimization of the dimensions of the soft actuator for the artificial midrib to minimize the working pressure at a tip force of 7.35 N, starting at 146.18 KPa and ending at 120.01 KPa. Optimal dimensions were found for *r* = 11 mm, *t*_2_ = 5 mm and *t*_3_ = 3.5 mm, corresponding to a minimum working pressure of 119.52 KPa.

### 5.2. Experimental Validation

Four sets of soft fiber-reinforced bending actuators with different dimensions were selected to validate the optimization results. Table 2 shows the geometrical parameters of the four sets of the selected soft actuator and the corresponding working pressure values. No. 1 is the initial parameter, No. 2 is the intermediate parameter in the optimization process, and No. 3 and No. 4 are the optimal parameters used in the artificial leaves and artificial midrib, respectively. No. 1, No. 2 and No. 3 are used to validate the optimization results of the soft actuator for the artificial leaves. No. 1, No. 2 and No. 4 are used to validate the optimization results of the soft actuator for the artificial midrib. Three soft actuators were fabricated and tested for each set. An air compressor (OTS Co., Ltd., Taizhou, China) and a pressure regulator (AirTAC Co., Ltd., Ningbo, China) generate constant working pressure. As shown in Figure 7b, an acrylic box was fabricated to restrain the bending angle of the soft actuator, a force sensor (CHINO SENSOR Co., Ltd., Bengbu, China) was placed in a groove on the bottom of the box, a weight was placed on the top of the box to hold the box in place. The proximal end of the soft actuator was fixed, and the distal end was placed on top of the force sensor, ensuring that the soft actuator was level and 5mm from the top of the box. Figure 7c,d show the experimental and numerical working pressure-tip force curves of selected actuators, where the tip force is the average value for a soft actuator with a certain set of dimensions under different working pressures. It can be seen that the experimental and numerical trends are generally consistent, but the experimental results require a larger working pressure to produce the same magnitude of tip force. This error is caused by hand-made. The soft actuator with optimum parameters produces the same tip force with minimal working pressure. At a tip force of 3.96 N, the required working pressures for the soft actuator using the optimum, intermediate and initial parameters are 98.03 KPa, 109.34 KPa and 116.35 KPa, respectively, and at a tip force of 7.35 N, the required working pressures for the actuator using the optimum, intermediate and initial parameters are 141.11 KPa, 150.51 KPa and 158.67 KPa, respectively.

Based on the optimization results and experimental validation, we used the soft actuators with geometrical parameters *r* = 11 mm, *t*_1_ = 1 mm, *t*_2_ = 9 mm, *t*_3_ = 3.5 mm and *p* = 4 mm to drive the artificial leaves and the soft actuator with geometrical parameters *r* = 11 mm, *t*_1_ = 1 mm, *t*_2_ = 5 mm, *t*_3_ = 3.5 mm and *p* = 4 mm to drive the artificial midrib.

## 6. Characterization of the AVFT

**Figure 8:**
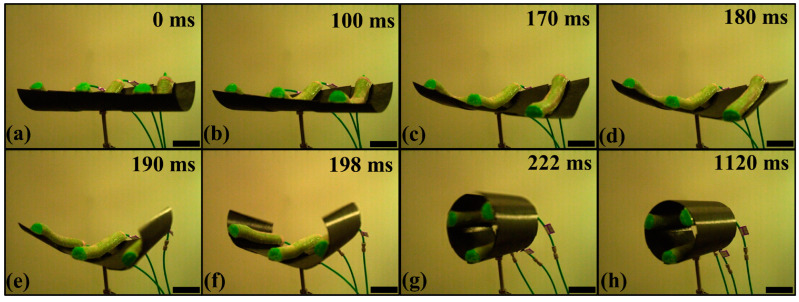
Closure sequence of the AVFT. (**a**–**c**) show the driving process with drive time of 170 ms, (**d**–**g**) show the snap process with snap time of 52 ms and (**h**) shows the stable closed state. Scale bars, 50 mm.

In order to obtain the closure time of the AVFT, a high-speed digital camera (VRI, Inc., Wayne, NJ, USA) with 500 frames per second was used to record the closure process. Three soft actuators of the AVFT in the open state were inflated simultaneously and slowly until they snapped rapidly to the closed state. After five measurements and taking the average of the testing data, it was determined that the minimum working pressure for the soft actuators to close the AVFT is 40 KPa. Then, 40 KPa of compressed air was supplied directly to three soft actuators to obtain the closure sequence of the AVFT through the high-speed digital camera. As shown in Figure 8, the closure process of the AVFT consists of a driving process and a snap process, in which the air compressor is closed during the snap process, and the AVFT only relies on its own bistability to deform. Figure 8a,c show the driving process of the AVFT. During the driving time of 170 ms, the curved soft actuators gradually straighten due to the increase of the working pressure in the air chamber, and the surfaces are gradually unfolded as normal forces are applied to the artificial leaves and artificial midrib along the interaction points. Once the accumulated total strain energy increases to a critical value, the AVFT will enter the rapid snap process. Figure 8c,g show the snap process of the AVFT, with the curvature of the right leaf changing first, followed by the snap of the left leaf. The difference in the critical trigger force of the leaves is the reason for the asynchronous snap. The artificial midrib, as a transitional section between two artificial leaves, always has a continuous curvature with the two artificial leaves; thus, its snap starts earliest and ends latest. The AVFT completely closes at 222 ms. The snap time is determined to be within 52 ms after several tests. The AVFT then oscillates a few times and finally rests at 1120 ms. Figure 8h shows the AVFT in the closed state (see Supplementary Information S2 for the video of the complete closure process of the AVFT).

We summarized the characterization values of the AVFTs based on bistable CFRP structures in the last decade, including reversibility, capture range, driving time and snap time, as shown in Table 3. Due to the introduction of the artificial midrib, the capture range of the AVFT is increased to 180°, which is much larger than the previous AVFTs. The shape memory alloy needs to be heated and transformed into austenite to drive the object. Similarly, the magnetic driving method also needs a certain period of time to increase the current to obtain a sufficient magnetic force. Due to the excellent responsiveness of the pneumatic actuation, the driving time is well below 1 s. For snap time, our AVFT is at least 35% faster than Kim’s AVFTs and at least 53.6% faster than Zhang’s AVFTs. The special series bistable CFRP structures increase the critical total strain energy of the AVFT, and the total strain energy is 0 when the AVFT is in the closed state (artificial leaves and artificial midrib are in the initial stable state). Therefore, the AVFT obtains higher potential energy difference, which can be converted into more kinetic energy, that is, less snap time. Due to the fact that the centers of curvature in the two stable states are on the same side of the antisymmetric laminated CFRP structure, conventional soft fiber-reinforced bending actuators cannot achieve reversibility like some AVFTs in the past, which is also one of our future improvement directions.

**Figure 9:**
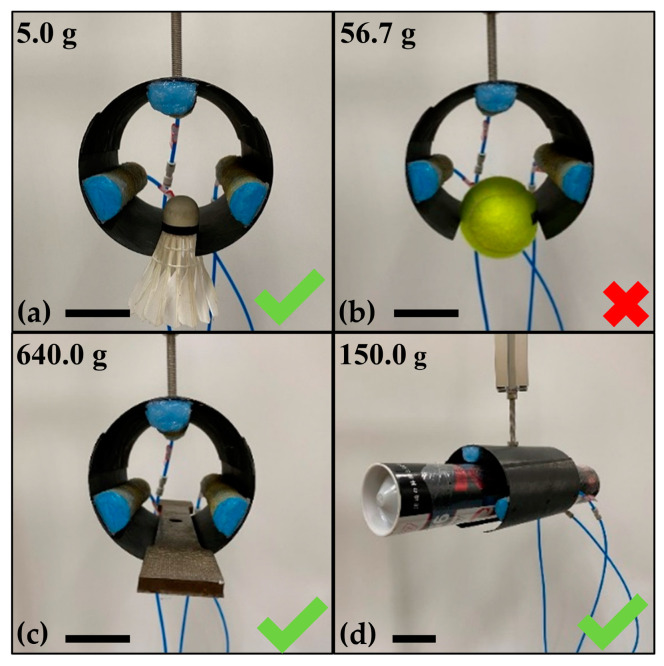
Three ways for the AVFT to grasp objects, where (**a**,**b**) are clamped grasping, (**c**) is supported grasping, and (**d**) is wrapped grasping. Scale bars, 50 mm.

The Venus flytrap can successfully capture the prey through the rapid closure motion and the tight closure state. To prove that the AVFT has a similar grasping performance to the real Venus flytrap, we showed three ways that the AVFT can be used as a soft gripper to grab objects. Note that the AVFT oscillates after the closure process; the grasping actions shown here are all in the stable stage after the oscillation. The first grasping way is called clamped grasping, which uses the resilience generated by the passive deformation of the bistable CFRP structure to grasp light and low-stiffness objects, such as a badminton weighing about 5.0 g in Figure 9a. However, for a spherical object such as a tennis weighing about 56.7 g as shown in Figure 9b, the AVFT has only two points of contact with it and therefore cannot grasp it successfully. The grasping way illustrated in Figure 9c is called supported grasping. Due to the excellent shape retention performance of the bistable CFRP structures, the AVFT can grasp objects with a certain weight by means of the normal constraining force of the bistable CFRP structures, and rely on friction to keep the objects stable, such as a metal block weighing 0.64 kg and 200 mm long. However, when using this grasping way to grasp heavier objects, a gap is created between the two artificial leaves, which cannot provide enough normal constraining force and causes grasping failure. When the cross-sectional area of the grasped object is large enough, such as a long cylindrical barrel shown in Figure 9d, the AVFT can rely on both the normal constraining force and resilience of the bistable CFRP structure to grasp, and this grasping way is called wrapped grasping. Since the soft actuators also provide a certain amount of friction, this grasping way is the most stable for grasping cylindrical objects compared to other grasping ways.

## 7. Conclusions

This paper describes a new bio-inspired pneumatic AVFT from the following aspects: theoretical prediction, design and manufacturing, experimentation, finite element analysis, optimization and characterization, which enables faster, flytrap-like closure movements and a 180° closure range at low working pressure and energy consumption. The AVFT without complex structures is composed of two artificial leaves and one artificial midrib made by bistable antisymmetric laminated CFRP structure, and three soft fiber-reinforced bending actuators. Inflating the soft actuators makes the artificial leaves and the artificial midrib snap. The bistable mechanism of the artificial leaves and the artificial midrib allows the AVFT to maintain a stable closed state without continuous energy input. A two-parameter model was used to prove that the selected antisymmetric laminated CFRP structure has bistable behavior, and the longitudinal curvature of the structure in the second stable state is only related to the transverse curvature in the initial stable state, the material properties of the CFRP, and the lay-up method. These conclusions provide theoretical guidance for the main part design of the AVFT. We designed an experiment without expensive equipment to obtain the critical trigger forces of 3.96 N and 7.35 N for the artificial leaves and the artificial midrib, and the numerical results matched well with the experimental results. To reduce the working pressure of the soft actuators, we optimized two groups of dimensions corresponding to the artificial leaves and the artificial midrib, using the minimum working pressure required to achieve the tip force equal to the critical trigger forces as the objective function, and carried out experiments to validate the reliability of the optimization. Finally, the minimum working pressure required to drive the AVFT to close was measured to be 40 KPa, and the driving time was 170 ms, with the required snap time being within 52 ms, which achieves a faster closure speed than the previous AVFTs. The AVFT has three ways to grasp objects, including supported grasping, clamped grasping and wrapped grasping, where grasping cylindrical objects using the wrapped grasping is the most stable. This novel AVFT is of great significance for the development of space satellites, soft grippers, and self-locking structures. Our future work has the following three points: (a) Continuing to optimize the AVFT to obtain reversibility, and exploring more application scenarios for various industrial demands; (b) Finding a more suitable bonding method or directly coupling the soft actuators with the bistable CFRP structures; (c) Exploring the service life of the AVFT, including the bistable CFRP structures (main part) and the soft actuators (driving part), is of great significance to the practical application of the AVFT.

## Figures and Tables

**Table 1:** The material properties of the T800/924C epoxy resin-based CFRP [65].

*E*_1_/GPa	*E*_2_/GPa	$\nu_{12}$	*G*_12_/GPa	*G*_13_/GPa	*G*_23_/GPa	*t*_ply_/mm
145	9.5	0.3	5	3.7	3.7	0.125

**Table 2:** Optimization numerical results with different parameter values and grouping of validation experiments.

Geometric Parameters/mm	No. 1	No. 2	No. 3	No. 4
	*r* = 10 *t*_1_ = 2 *t*_2_ = 10 *t*_3_ = 2.5 *p* = 4	*r* = 10.4 *t*_1_ = 1.6 *t*_2_ = 7 *t*_3_ = 2.7 *p* = 4	*r* = 11 *t*_1_ = 1 *t*_2_ = 9 *t*_3_ = 3.5 *p* = 4	*r* = 11 *t*_1_ = 1 *t*_2_ = 5 *t*_3_ = 3.5 *p* = 4
Working pressure/KPa when tip force = 3.96 N	95.49	89.13	76.56	-
Working pressure/KPa when tip force = 7.35 N	146.18	135.25	-	119.52

**Table 3:** Comparison with previous AVFTs based on bistable CFRP structures.

The AVFTs Based on Bistable CFRP Structures	Lay-Up	Actuation	Reversibility	Capture Range/° *	Driving Time/s	Snap Time/ms
Kim, S. et al. (2010) [6]	[0°/90°]	SMA	No/manually	≈90	null	80
Kim, S. et al. (2011) [7]	[0°/90°]	SMA	Yes	≈150	≤2.86	100
Kim, S. et al. (2014) [8]	[0°/90°]	SMA	Yes	≈150	≥4.4	≤100
Zhang, Z. et al. (2016) [9]	[45°/−45°/45/°−45°]	Magnetic	No/manually	≈150	1.32	200
Zhang, Z. et al. (2019) [10]	[45°/−45°/45/°−45°]	Magnetic	No/manually	≈120	null	112
Zhang, Z. et al. (2022) [33]	[0°/90°]	Pneumatic	Yes	≈60	null	170
Our AVFT	[45°/−45°/45/°−45°]	Pneumatic	No/manually	180	0.17	52

null: The author does not mention the item. *: Approximate values obtained from the physical diagram in the article.

## Data Availability

The original contributions presented in the study are included in the article, and further inquiries can be directed to the corresponding author.

## Supplementary Materials

The following supporting information can be downloaded at: https://www.mdpi.com/article/10.3390/biomimetics8020181/s1, Supplementary Information S1: Design of a Bistable Artificial Venus Flytrap Actuated by Low Pressure with Larger Capture Range and Faster Responsiveness [72,73]; Figure S1: Deformation of the cross section of the structure; Figure S2: Diagram of the cured stacking arrangement of the bistable antisymmetric laminated carbon fiber-reinforced prepreg (CFRP) structure; Figure S3. The stress-strain curve for the silicone rubber (Smooth-on 950) based on experimental test data and theoretical model data. The second order polynomial model fits the experimental test data best with the coefficients determined as *C10* = −0.01198, *C20* = −0.004238, *C01* = 0.1305, *C02* = 0.2294 and *C11* = −0.004517.; Figure S4: The percentage contribution of the five geometric parameters to the working pressure required when the tip force equals the critical trigger force, with blue indicating positive effects and red negative effects.; Figure S5. Optimization iteration of soft fiber-reinforced bending actuators, where (a) and (b) correspond to artificial leaves and (c) and (d) correspond to artificial midrib. Yielding the optimal geometric dimensions of *r* =11 mm, *t*_2_ = 9 mm, *t*_3_ = 3.5 mm and *r* = 11 mm, *t*_2_ = 5 mm, *t*_3_ = 3.5 mm, respectively.; Video S1: Supplementary Information S2.
