# Supplementary material for: Design of a Bistable Artificial Venus Flytrap Actuated by Low Pressure with Larger Capture Range and Faster Responsiveness
Source: Biomimetics (Basel). 2023 Apr 26;8(2):181. doi: 10.3390/biomimetics8020181 (PMC10204398; doi:10.3390/biomimetics8020181)
Supplement: Supplementary file 1 [file biomimetics-08-00181-s001.zip › biomimetics-2320272-Supplementary Information I.pdf]

Supplementary Information I

Design of a Bistable Artificial Venus Flytrap Actuated by Low Pressure with Larger Capture Range and Faster Responsiveness

Junchang Yang, Fenghui Wang * and Yongjun Lu

Bio-inspired and Advanced Energy Research Center, School of Mechanics, Civil, Engineering and Architecture, Northwestern Polytechnical University, Xi'an 710129, P. R. China; jcyang@mail.nwpu.edu.cn (J.Y.); luyongjun@mail.nwpu.edu.cn (Y.L.)

* Correspondence: fhwang@nwpu.edu.cn

Note 1. Extensional bending model [28]

The Extensional bending model proposed by Iqbal and Pellegrino [28] for antisymmetric laminated carbon fiber-reinforced prepreg (CFRP) structure is based on the Classical Lamination Theory and the principle of minimum potential energy, which includes expressions for the bending and stretching strain energies. Geometric parameters of the antisymmetric laminated CFRP structures are illustrated in Figure 1. In the initial stable state, the radius of the cross-section arc is R_1, and the subtended angle is β. In the second stable state, the radius of the cross-section arc is R_2. The two intermediate states of the snap process of structure, which can be described by transverse curvature κ_y and longitudinal curvature κ_x. Before deriving the expression, the following assumptions need to be specified:

a) Laminates are within the assumption of linear elasticity and in the state of plane stress.
b) Follow the Kirchhoff-Love assumption.
c) The twist curvature $\kappa_{xy} = 0$.
d) The coupling stiffness $\boldsymbol{B} = 0$.
e) The longitudinal curvature κ_x and the transverse curvature κ_y are uniform throughout the structure.

The bending and stretching strain energies of antisymmetric laminated CFRP structures are deduced respectively as follows (The ***ABD*** matrix is introduced in Equation (1) of the article):

(1) The bending strain energy

The bending strain energy per unit area in a flat, antisymmetric laminated structure can be expressed as

$$u_b = \frac{1}{2}\begin{bmatrix}\kappa_x & \kappa_y & \kappa_{xy}\end{bmatrix}\boldsymbol{D}\begin{bmatrix}\kappa_x \\ \kappa_y \\ \kappa_{xy}\end{bmatrix}, \tag{S1}$$

Since $\kappa_{xy} = 0$, Equation (S1) can be expanded as

$$u_b = \frac{1}{2}\left(D_{11}\kappa_x^2 + 2D_{12}\kappa_x\kappa_y + D_{22}\kappa_y^2\right), \tag{S2}$$

For a structure with initial transverse curvature $1/R_1$, Equation (S2) can be rewritten as

$$u_b = \frac{1}{2}\left[D_{11}\kappa_x^2 + 2D_{12}\kappa_x\left(\kappa_y - \frac{1}{R_1}\right) + D_{22}\left(\kappa_y - \frac{1}{R_1}\right)^2\right], \tag{S3}$$

Then the bending strain energy per unit length in an antisymmetric laminated structure can be expressed as

$$U_b = \int_A u_b dA = \frac{\beta R_1}{2}\left[D_{11}\kappa_x^2 + 2D_{12}\kappa_x\left(\kappa_y - \frac{1}{R_1}\right) + D_{22}\left(\kappa_y - \frac{1}{R_1}\right)^2\right]. \tag{S4}$$

(2) The stretching strain energy

The stretching strain energy per unit area in an antisymmetric laminated structure can be expressed as

$$u_s = \frac{1}{2}\begin{bmatrix}\varepsilon_x^0 & \varepsilon_y^0 & \gamma_{xy}^0\end{bmatrix}\boldsymbol{A}\begin{bmatrix}\varepsilon_x^0 \\ \varepsilon_y^0 \\ \gamma_{xy}^0\end{bmatrix} \tag{S5}$$

The deformed cross section of the structure is shown in Figure S1. z is the distance from any point P on the cross section to the neutral axis of the structure. Neutral axis passes through the centroid G of the cross section. d is the distance from the centroid G to the origin O and can be expressed as

$$d = \frac{2\int_0^{\frac{1}{2}\beta R_1\kappa_y}\frac{1}{\kappa_y}\cos\theta\cdot\frac{1}{\kappa_y}d\theta}{\beta R_1} = \frac{2\sin\left(\beta R_1\kappa_y/2\right)}{\beta R_1\kappa_y^2}, \tag{S6}$$

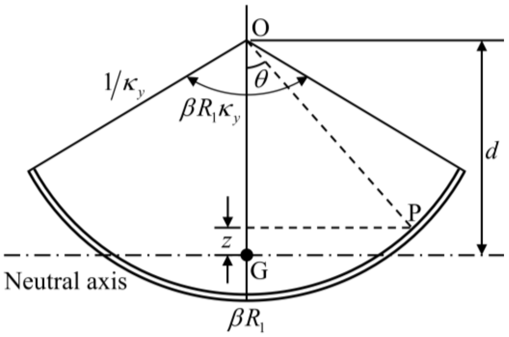

Figure S1. Deformation of the cross section of the structure

Then, longitudinal strain ε_x can be expressed as

$$\varepsilon_x^0 = z\kappa_x = \left(d - \frac{\cos\theta}{\kappa_y}\right)\kappa_x = \left(\frac{2\sin\left(\beta R_1 \kappa_y/2\right)}{\beta R_1 \kappa_y^2} - \frac{\cos\theta}{\kappa_y}\right)\kappa_x, \tag{S7}$$

As $\varepsilon_y^0 = \gamma_{xy}^0 = 0$, substituting Equation (S7) into Equation (S5) gives

$$u_s = \frac{1}{2}A_{11}\left(\varepsilon_x^0\right)^2 = \frac{A_{11}}{2}\left[\frac{2\sin\left(\beta R_1 \kappa_y/2\right)}{\beta R_1 \kappa_y^2} - \frac{\cos\theta}{\kappa_y}\right]^2 \kappa_x^2, \tag{S8}$$

Then integrate the cross-section of the structure to obtain the stretching strain energy per unit length in an antisymmetric laminated structure

$$\begin{aligned} U_s &= \int_A u_s dA = 2\int_0^{\frac{1}{2}\beta R_1 \kappa_y} u_s \cdot \frac{1}{\kappa_y} d\theta \\ &= \frac{A_{11}}{2}\left[\frac{\beta R_1}{2}\left(\frac{\kappa_x^2}{\kappa_y^2}\right) + \frac{\sin\left(\beta R_1 \kappa_y\right)}{2}\left(\frac{\kappa_x^2}{\kappa_y^3}\right) - \frac{4\sin^2\left(\beta R_1 \kappa_y/2\right)}{\beta R_1}\left(\frac{\kappa_x^2}{\kappa_y^4}\right)\right], \end{aligned} \tag{S9}$$

Notice that

$$\lim_{\kappa_y \to 0} U_s = 0 \tag{S10}$$

Therefore

$$U_s = \begin{cases} \dfrac{A_{11}}{2}\left[\dfrac{\beta R_1}{2}\left(\dfrac{\kappa_x^2}{\kappa_y^2}\right) + \dfrac{\sin\left(\beta R_1 \kappa_y\right)}{2}\left(\dfrac{\kappa_x^2}{\kappa_y^3}\right) - \dfrac{4\sin^2\left(\beta R_1 \kappa_y/2\right)}{\beta R_1}\left(\dfrac{\kappa_x^2}{\kappa_y^4}\right)\right] & \left(\kappa_y \neq 0\right) \\ 0 & \left(\kappa_y = 0\right) \end{cases}. \tag{S11}$$

(3) The total strain energy

Neglecting coupling effects, the total strain energy in a unit length of antisymmetric laminated structure can be obtained

$$U = U_b + U_s, \tag{S12}$$

If the lay-up method and material properties of the structure are known, the total strain energy is only determined by two parameters κ_y and κ_x. According to the principle of minimum potential energy, the two stable states of the antisymmetric laminated structure correspond to the two local minima of the total strain energy, respectively. Therefore, only by solving

$$\begin{cases} \dfrac{\partial U}{\partial \kappa_x} = 0 \\ \dfrac{\partial U}{\partial \kappa_y} = 0 \end{cases}, \tag{S13}$$

the corresponding transverse and longitudinal curvatures of antisymmetric laminated structure in two stable states can be obtained.

Note 2. Finite Element Analysis for snap process of the selected bistable antisymmetric laminated CFRP structure

We used Abaqus (Dassault Systèmes, Aachen, Germany) to simulate the snap process of the bistable antisymmetric laminated CFRP structure. The finite element geometry model is the same as the bistable CFRP structure in the initial stable state, and the geometric center of the model was used as a fixed point. The material properties of the CFRP are shown in Table 1, and the lay-up is [45°/−45°/45°/−45°]. 4-node reduced integration curved shell elements (Abaqus element type S4R) with a global mesh cell size of 1mm was selected for the simulation. The fixed point of the model was constrained by "ENCRETE" boundary condition. Applying a sufficiently large bending moment to two straight edges of the model to drive the structure to snap, then unloading the bending moment to obtain the geometric configuration of the structure in the second stable state. Choosing "Static, General" as the type of all created step. Considering the non-linear deformation of the bistable CFRP structure during the snap process, we turned on the "Nlgeom" option. Checking "Automatical stabilization" and selecting the "Damping factor", keeping the default factor of 0.0002 to make the calculation results converge more easily. The method carries out a pseudo-dynamic simulation as soon as a negative pivot is detected during the inversion of the stiffness matrix, and introduce fictitious nodal masses and a small amount of numerical damping to stabilize the snap [72].

Note 3. Fabrication of the bistable antisymmetric laminated CFRP structure

The curvature of the bistable antisymmetric laminated CFRP structure in the initial stable state can be controlled by curing on a mold with a specific curvature, thus we manufactured a curved jig plate with a curvature of 20.94 m^{-1} to obtain a bistable CFRP structure with a radius of curvature R of 150/π mm. The plate was heat treated to prevent deformation caused by high temperatures. In this study, we used the T800/924C epoxy resin-based CFRP with a ply thickness of 0.125 mm (GW COMPOS Co., Ltd., Weihai, China). The CFRP was cut into rectangular sections measuring 100 × 150 mm and 150 × 150 mm, while ensuring that the carbon fiber direction is 45° from the edges. Figure S2 shows a diagram of the cured stacking arrangement of the bistable CFRP structure, the plate was first evenly sprayed with release agent, and then the CFRP was laid on the mold in a lay-up of [45°/−45°/45°/−45°], ensuring that the long straight edge of the CFRP is parallel to the axis of curvature of the plate groove. We laid a peelable film, a partition film and a ventilated felt on the laminate in sequence, where the peelable film is used to create a smooth surface and the partition film makes the laminate easier to be removed from the mold. Finally, the vacuum bag was wrapped around all the material including the plate and sealed with a high-temperature resistant sealing tape. The vacuum inside the bag was maintained during curing and the curing temperature was increased from room temperature 25 °C to 180 °C with a curing time of 2 hours. After curing and cooling down to room temperature, we obtained two types of bistable antisymmetric laminated CFRP structures with different central angles of θ_1 = 120° and θ_2 = 180° as artificial leaves and artificial midrib respectively.

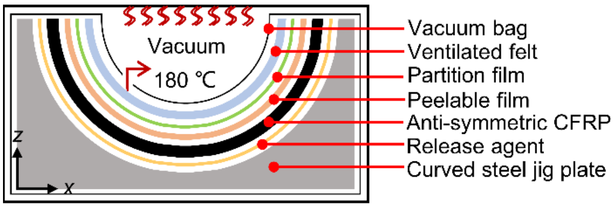

Figure S2. Diagram of the cured stacking arrangement of the bistable antisymmetric laminated carbon fiber-reinforced prepreg (CFRP) structure.

Note 4. Fabrication of the soft fiber-reinforced bending actuator

Smooth-Sil 950 silicone rubber (Smooth-on, Inc., Macungie, PA, USA) with a hardness of 50 A was chose for the elastic body. A piece of paper as a strain limiting layer was adhered to the flat surface of the elastic body. Kevlar fibers with a wire diameter of 0.4 mm were then wound along the indentation on the elastic body surface and fixed with Ausbond 441 silicone adhesive (Ausbond Co., Ltd., Tai'an, China). The fabrication process follows a traditional cast molding method [50,51]. The molds consisting of a top mold, a base mode, a half round rod and a mold cap, all were designed in Catia (Dassault Systèmes, Aachen, Germany) and manufactured using stereo lithography appearance, with the 3D printing material being ABS-like resin.

Note 5. Finite Element Analysis for the critical trigger forces of the artificial leaves and the artificial midrib

The pre-processing of the finite element analysis of the critical trigger force is similar to the previous section, except that the boundary conditions were adjusted to be the same as in the experiment. The finite element geometry model is the same as the bistable CFRP structure in the initial stable state, cutting the model to obtain a fixed point and two loading areas, which are in the same position as the experiment. We divided the analysis step of the finite element model into four steps. In step 1, we applied bending moments to two straight edges of the bistable CFRP structure, caused it to snap from the initial stable state to the second stable state. In step 2, the bending moment was unloaded and the structure was finally kept in the second stable state. In step 3, pressure was applied at both loading areas, and the magnitude of pressure increasing from the critical trigger force obtained experimentally until the model was able to complete the snap process. In step 4, the pressure was unloaded and the model returned to its initial stable state.

Note 6. Finite Element Analysis for the tip force of the soft fiber-reinforced bending actuator

We use Abaqus to obtain a numerical relationship between the tip force generated by the soft fiber-reinforced bending actuator at a bending angle of 0° and the working pressure. The model consists of a soft actuator, a top restraint and a bottom restraint. The distance between the top restraint and the top of the soft actuator is 5 mm and the bottom restraint is in contact with the distal end of the soft actuator. As shown in Figure S3, the second order polynomial model with the coefficients $C10 = -0.01198$, $C20 = -0.004238$, $C01 = 0.1305$, $C02 = 0.2294$ and $C11 = -0.004517$ was determined the hyperelastic constitutive model for the silicone rubber (Smooth-Sil 950) used to fabricate the elastic body of the soft actuator through uniaxial tensile tests. Kevlar fibers as the flexible inextensible fibers and a piece of paper as the strain limiting layer were modelled as linear elastic models with material parameters of $E = 31076$ MPa, $\nu = 0.36$ and $E = 1.2$ GPa, $\nu = 0.237$ respectively [73]. The elastic body of the soft actuator was modelled as solid tetrahedral quadratic hybrid elements (Abaqus element type C3D10H), the flexible inextensible fibers were modelled using quadratic beam elements (Abaqus element type B32) and the strain limiting layer was modelled using 8-node reduced integration curved shell elements (Abaqus element type S8R). To improve computational efficiency, both the top and bottom constraints were set as discrete rigid bodies and used quartic rigid elements (Abaqus element type R3D4). "ENCASTRE" boundary conditions were applied to the proximal end of the soft actuator and to the reference points of the top constraint and the bottom constraint. The flexible inextensible fibers and the strain-limiting layer were connected to the elastic body by tie restraints. The working pressure was loaded linearly with step time onto the surface of the air chamber in the soft actuator with a magnitude of 200 KPa. Due to the non-linear deformation of the actuator, the "Nlgeom" option was switched on. As the working pressure increases, the bottom restraint is subjected to the tip force exerted by the soft actuator, which can be extracted from the reference point of the bottom restraint.

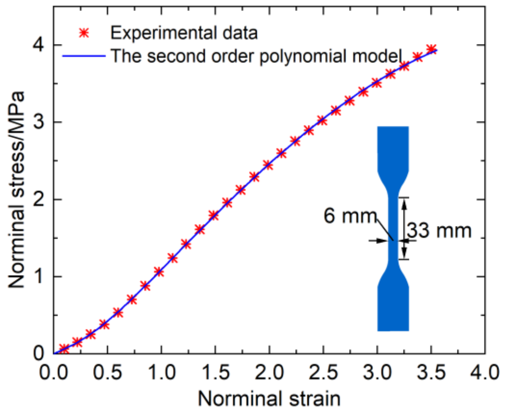

Figure S3. The stress-strain curve for the silicone rubber (Smooth-on 950) based on experimental test data and theoretical model data. The second order polynomial model fits the experimental test data best with the coefficients determined as $C10 = -0.01198$, $C20 = -0.004238$, $C01 = 0.1305$, $C02 = 0.2294$ and $C11 = -0.004517$.

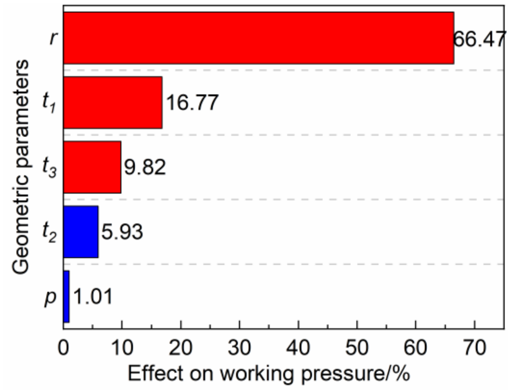

Figure S4. The percentage contribution of the five geometric parameters to the working pressure required when the tip force equals the critical trigger force, with blue indicating positive effects and red negative effects.

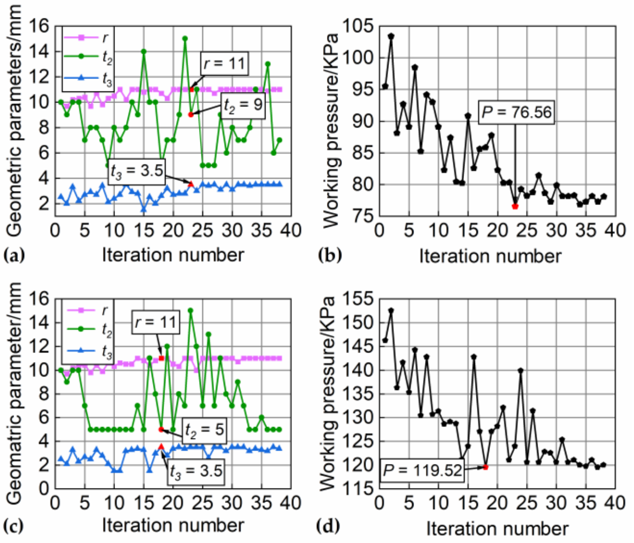

Figure S5. Optimization iteration of soft fiber-reinforced bending actuators, where (a) and (b) correspond to artificial leaves and (c) and (d) correspond to artificial midrib. Yielding the optimal geometric dimensions of r =11 mm, t_2 = 9 mm, t_3 = 3.5 mm and r = 11 mm, t_2 = 5 mm, t_3 = 3.5 mm, respectively.
